# The 5x1 DAFNE study protocol: a cluster randomised trial comparing a standard 5 day DAFNE course delivered over 1 week against DAFNE training delivered over 1 day a week for 5 consecutive weeks

**DOI:** 10.1186/1472-6823-12-28

**Published:** 2012-11-08

**Authors:** Jackie Elliott, Julia Lawton, David Rankin, Celia Emery, Mike Campbell, Simon Dixon, Simon Heller

**Affiliations:** 1 Academic Unit of Diabetes, Endocrinology & Metabolism, Department of Human Metabolism, The University of Sheffield, The Medical School, Beech Hill Road, Sheffield, S10 2RX, UK; 2 Centre for Population Health Sciences, The University of Edinburgh, Medical School, Teviot Place, Edinburgh, EH8 9AG, UK; 3 DAFNE NIHR Project Office, 11 Broomfield Rd, Sheffield, S10 2SE, UK; 4 School of Health and Related Research, University of Sheffield, Regent Court, 30 Regent Street, Sheffield, S1 4DA, UK

**Keywords:** Type 1 diabetes, Education, Teaching, Training programs

## Abstract

**Background:**

Structured education programmes are now established as an essential component to assist effective self-management of diabetes. In the case of Type 1 diabetes, the Dose Adjustment For Normal Eating (DAFNE) programme improves both glycaemic control and quality of life. Traditionally delivered over five consecutive days, this format has been cited as a barrier to participation by some patients, such as those who work full-time. Some centres in the UK have organised structured education programmes to be delivered one day a week over several consecutive weeks. This type of format may add benefit by allowing more time in which to practice skills between sessions, but may suffer as a result of weaker peer support being generated compared to that formed over five consecutive days.

**Methods/design:**

We aim to compare DAFNE delivered over five consecutive days (1 week course) with DAFNE delivered one day a week over five weeks (5 week course) in a randomised controlled trial. A total of 213 patients were randomised to attend either a 1 week or a 5 week course delivered in seven participating centres. Study outcomes (measured at baseline, 6 and 12 months post-course) include HbA_1c_, weight, self-reported rates of severe hypoglycaemia, psychosocial measures of quality of life, and cost-effectiveness. Generalisability was optimised by recruiting patients from DAFNE waiting lists at each centre, and by mailing eligible patients from hospital clinic lists. The inclusion and exclusion criteria were identical to those used to recruit to a standard DAFNE course (e.g., HbA_1c_ <12%, with no lower limit). Qualitative interviews were undertaken with a sub-sample of n=30 patients and their course educators (n=11) to help understand and interpret differences and similarities in outcomes between the two arms, and to identify logistical problems and unanticipated issues arising from the adaptation and delivery of a 5 week course.

**Discussion:**

This trial has been designed to test the hypothesis that the benefits of delivering a structured education programme over 5 weeks are comparable to those observed after a 1 week course. The results of the trial and the qualitative sub-study will both inform the design and delivery of future DAFNE courses, and the development of structured education programmes in other fields of medicine.

**Trial Registration:**

Clinicaltrials.gov NCT01069393

## Background

The DAFNE (Dose Adjustment for Normal Eating) course is a 1 week structured education course teaching skills in insulin use and dietary freedom to individuals with Type 1 diabetes, which was originally adapted from a German inpatient programme [1]. The original DAFNE trial, published in 2002, demonstrated that a dose adjustment for normal eating approach led to improved glycaemic control and quality of life without increases in severe hypoglycaemia, benefits which lasted for at least 1 year [2]. DAFNE is now a nationally and internationally delivered evidence-based, quality assured programme [3], delivered in 76 centres across the UK and as well as in Ireland, Australia (Oz DAFNE), New Zealand, Kuwait and Singapore.

The development of structured education programmes, such as DAFNE has had a major impact on the treatment of Type 1 diabetes in the UK. The Department of Health [4] now requires structured education programmes to be offered to all individuals with Type 1 diabetes in England and Wales, and most secondary care centres offer some form of skills training to adults with Type 1 diabetes. However, the format of non-DAFNE programmes currently offered to patients varies widely in terms of duration, number of sessions, level of quality control and designs are not evidence based. It has been suggested that it may not always be easy for some people to find the time to attend an intensive 1 week course, patients cite difficulties due to work obligations or arranging child-care. This may, in part, explain the observation that around 50% of adults with Type 1 diabetes attending centres which run DAFNE training have not yet received this type of education. Meanwhile, many non-DAFNE structured education courses are delivered on a one day a week basis for between 2 and 8 weeks [5] which may have improved access, although in the absence of any definitive data this is hypothetical. There has been one randomised control trial examining the effect of a shorter period of structured education (2.5 days) over 6 weeks in a single centre, but no improvements in biomedical outcomes were observed [6].

Some non-DAFNE centres have reported outcomes during routine care in self-selected individuals which are comparable to those reported in the original DAFNE trial [7]. Yet, since observational data of this type are prone to bias it is unclear whether such an extended approach to skills training is as effective as that provided on a 1 week intensive course. This randomised control trial is designed to test the hypothesis that the benefit of a structured education programme, with the identical contact time, is the same whether delivered over 1 or 5 weeks.

There are theoretical reasons why the two approaches might lead to different outcomes. A 1 week model might lead to stronger peer support developing over the week that could help to improve incorporation of self-management skills. Alternatively, a course delivered over 5 weeks might allow patients to gain more practice in self-management skills during the weeks between education sessions, thus facilitating integration of its principles into everyday life, and hence enhance sustainability. However, the potentially weaker peer support on a 5 week course may lead to a higher drop-out rate, with less participants completing the full 5 days of skills training.

Establishing whether DAFNE delivered at weekly intervals is as effective as delivering it over 5 consecutive days will help guide and inform decisions about future course delivery. Potentially it could also allow more individuals with Type 1 diabetes to receive structured education and increase patient choice. As qualitative work can provide important insights into trial outcomes [8], a qualitative sub-study was conducted in which we drew upon people’s understandings and experiences, by interviewing both educators and course participants. We also surveyed participants at the end of the trial asking whether they would prefer future structured education programmes to be delivered over 1 week or a more extended period of 5 weeks. Included in this survey were questions enquiring as to current self-management strategies.

## Methods/design

The DAFNE 5x1 day RCT is a multi-centre randomised trial comparing 2 different methods of delivering the DAFNE programme in the UK.

The objectives:

1) To explore whether the outcomes of a structured education programme with identical contact time are comparable whether delivered over 1 or 5 weeks.

2) To examine the cost effectiveness of a DAFNE course delivered either over 1 or 5 weeks.

3) To calculate how many patients choose to receive structured education programmes over 1 or 5 weeks, by conducting a preference survey.

4) The aims of the qualitative sub-study were

a) Help understand and interpret any differences and similarities in biomedical and psychological outcomes between patients attending DAFNE courses delivered over 1 or 5 weeks.

b) Identify any logistical problems and unanticipated issues arising from the adaptation and delivery of DAFNE as a course spread over 5 weeks.

5) Provide recommendations for the timing, content and delivery of future DAFNE courses.

Ethical approval was obtained from the Derbyshire Research Ethics Committee (09/H0401/91). Written informed consent was obtained from all trial participants, after they had had sufficient time to consider the Patient Information Sheet and had any questions pertaining to the trial answered. Additional written informed consent was obtained from participants in the qualitative sub-study. The study is registered with the local Research and Development department of each study centre and Sheffield Teaching Hospitals NHS Foundation Trust is acting as the sponsor.

Funding was secured as part of a NIHR programme grant for applied research – Improving management of Type 1 diabetes in the UK: the DAFNE programme as a research test-bed (application number RP-PG-0606-1184).

### Recruitment procedures

Secondary care centres with experience in delivering high quality structured education using DAFNE participated in the trial. When invited in 2009, seven out of a possible 74 DAFNE centres volunteered to take part. All eligible participants on the DAFNE waiting list received a written invitation to join the study from their local diabetes team. After indicating initial interest they were contacted by a member of the local research team or educator who provided further information, which, in some centres, included an invitation to an information evening, prior to obtaining informed consent. Participants were informed of a pair of course dates (a one week and a 5 week course) for which they would need to be available. After obtaining consent, the participants were randomised to either attend one or other format of course, i.e., either a 1 week or 5 week course. Recruitment took place between May 2010 and April 2011. When consent was obtained, patients were also asked to indicate whether their contact details could be made available to the qualitative research team in order that they might be approached to take part in the qualitative sub-study. Those who were approached were given further written information about the qualitative study and asked to opt-in using a separate consent form.

The *inclusion criteria* were as follows: Adults with Type 1 diabetes for at least 6 months, aged 18 – 80 y, HbA_1c_ <12%, willing to undertake intensive insulin therapy, with multiple self-monitoring of blood glucose (SMBG), carbohydrate counting, insulin self-adjustment and no strong views on attending a 1 week course or a 5 week course. Participants needed to agree to randomisation regardless of preference, having not yet completed DAFNE training or equivalent.

The *exclusion criteria* were as follows: severe diabetic complications (these include renal replacement therapy, severely impaired vision), inability to communicate in English, strong preference for either a 1 week or a 5 week course, severe needle phobia, unenthusiastic about undertaking SMBG and insulin self-adjustment, or inability to give informed consent.

### Sample size

Using a non-inferiority design, and based on clinically relevant difference of HbA_1c_ of 0.5%, a standard deviation of 1.5 and groups of 7–8 participants per randomisation group, for 80% power at one-sided 5% significance 150 participants were required to complete the study procedures. Allowing for a 10% drop-out from randomisation to completion of the trial, it was anticipated that we would require 166 subjects to be randomised in the study. Thus we aimed to run 24 courses (12 courses per arm). Each centre was required to recruit 32 patients to enable 4 courses (two standard DAFNE and two 5 week) to be run with 8 participants on each. However, during the recruitment phase of the trial it became apparent that the drop-out rate between randomisation and course participation was higher than had been anticipated, at over 11%, hence one centre ran an extra pair of courses.

**Figure 1:**
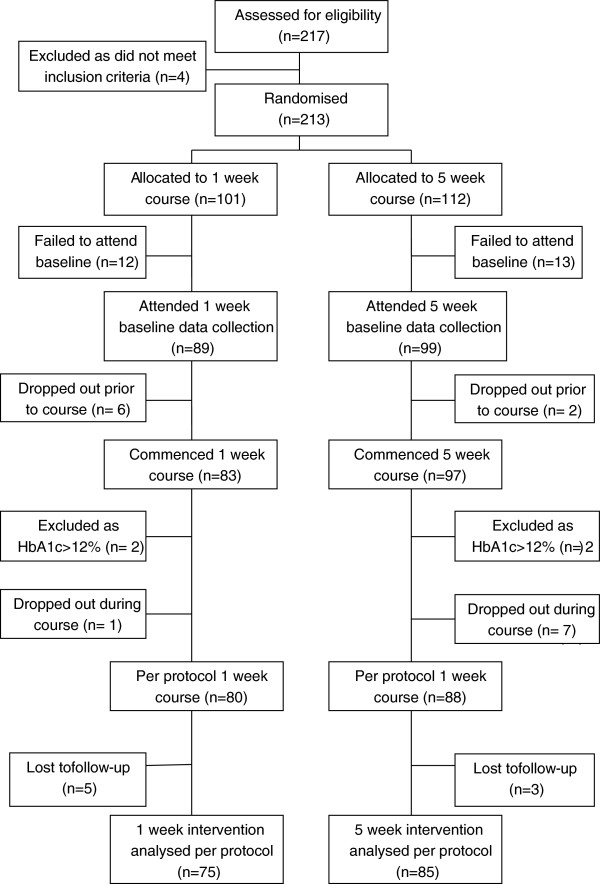
Flow diagram of the participant progress through the phases of the 5x1 randomised control trial.

The flow of patients through the trial is shown in Figure 1, this figure follows the CONSORT approach, as will future reporting of the trial results [9].

### Randomisation

After consent and completion of baseline data collection, the participants were individually randomised using a random block size, stratified by centre and a web-based remote randomisation system into either a 1 week course or a 5 week course. The allocation sequence was generated by MJC using a computer program called RANDOLOG (University of Southampton). The centres sent a list of participant study numbers to CE, the project manager, who performed the blinded randomisation and informed the centres of the results.

### The intervention

Patients were randomised to either:

• 1 week - The standard DAFNE 1 week course, or

• 5 weeks - The DAFNE course delivered on one day a week over 5 consecutive weeks

Although pragmatic in design, in order to try to reduce bias, each Centre’s 1 week and 5 week courses were delivered by the same two DAFNE educators, so that the skills, enthusiasm or teaching style of an educator did not favour one arm over the other. As defined for standard DAFNE courses, a participant was deemed to have completed the course if they attended for at least 4 full days of the 5 day course. Where a participant missed a day, attempts were made by the educators to cover missed material on an individual basis also as per normal DAFNE procedures. If a course could not be filled with trial participants, individuals undergoing DAFNE training but not consented to the trial (‘fillers)’ were included so as to maintain the normal dynamics of DAFNE group education.

### 1 week - The standard DAFNE 1 week DAFNE course

Each course took place over five consecutive days and was delivered to groups of 5–8 adults. The curriculum uses a progressive modular based structure to improve self-management in a variety of medical and social situations. The curriculum is designed to deliver key learning topics at the appropriate time within the programme. In this way, knowledge and skills are built up throughout the week with active participant involvement and problem solving as key methods of learning. The key modules are: what is diabetes; food and diabetes; insulin management; management of hypoglycaemia; sick day rules. Learning objectives for each day and each session are clearly identified and educators have instructions on session preparation and teaching materials. Lesson plans give guidance on timing and a student activity section serves to give an idea of expected responses. Each meal and snack is used as an opportunity to practise carbohydrate estimation and insulin dose adjustment.

### 5 weeks - The DAFNE course delivered on one day a week over 5 consecutive weeks

The curriculum was adapted from the standard course described above. This process was informed by qualitative interviews performed during an earlier mixed methods study of the DAFNE programme, in which patient and educator views were solicited as to how the curriculum could be adapted for a 5 week course. Four experienced DAFNE educators met with the qualitative researchers to exchange ideas and then further refinements were communicated within the group via e-mail. The contents of the two curricula were identical in terms of educational content and skills training although we made some minor changes to accommodate the needs of those attending over 5 weeks. Thus rather than providing a workbook on each day of the course, those attending 1 week courses received an entire workbook at the outset. The hope was that this would encourage participants to attend each week to gain more information and thereby reduce the drop-out rate. Also, blood glucose diaries were modified to allow for additional space to record to events / physical activity in more detail. The adapted curriculum was tested in 3 pilot courses, at 2 different centres, prior to commencement of the main trial. As a result of the pilot courses minor modifications were made to two of the sessions.

### Follow up

Patients attending both 1 week and 5 week courses were invited to a 6 week post course group-based meeting lasting 1–2 hrs to review DAFNE principles (a standard practice for regular DAFNE course attendees). At 6 and 12 months post the last day of the course, both groups were due to attend for measurement of weight, recording of hypoglycaemic episodes, collection of blood for HbA_1c_ and blood biochemistry, and completion of psychosocial questionnaires. Interviews for the qualitative sub-study were conducted during the week after courses had concluded, with follow-up interviews six months later.

### Outcomes

We measured outcomes on two occasions primarily to examine the time of maximum effect, and also because repeated measures improves the power to test the main objective.

The Primary outcome was:

Change in HbA_1c_ from baseline at 6 and 12 months. HbA_1c_, a measurement of glycosylated haemoglobin, is regarded as the gold standard measure of glycaemic control reflecting overall blood glucose values over the previous 8–12 weeks. There is a strong relationship between HbA_1c_ and the risk of developing long-term diabetic complications and it is accepted as a reasonable surrogate for long-term outcomes in individuals with diabetes. HbA_1c_ was measured at local laboratories DCCT aligned (as per Diabetes Control and Complications Trial [10]).

The Secondary Outcomes were:

Change in HbA_1c_ from baseline at 6 and 12 months for those with a baseline HbA_1c_ ≥7.5%, This is because some of the participants with low HbA_1c_s would be aiming to reduce the number of episodes of hypoglycaemia, as opposed to further reducing their HbA_1c_.

Biomedical: eGFR, and lipids (including HDL cholesterol), using local labs at baseline and 12 months as part of routine clinical monitoring.

Hypoglycaemia: Number of episodes of severe hypoglycaemia using a standard definition of an episode leading to cognitive impairment sufficient to cause either coma or requiring the assistance of another person to recover. (The number of severe episodes is considered to be reliably recalled by patients for up to 1 year. [11])

Psychosocial:

• Quality of life: Quality of life (QoL) is assessed using both the diabetes-specific quality of life scale (DSQOLS [12]), and the generic measures of QoL, SF-12 [13], and EQ-5D [14].

• Emotional Well-being: is assessed using the Hospital Anxiety and Depression Scale (HADS; [15]), the diabetes-specific Problem Areas in Diabetes Scale (PAID [16]).

• Self-efficacy: Diabetes-specific self-efficacy is assessed using the Confidence in Diabetes Self-Care measure [17] a validated, reliable measure for people with type 1 diabetes.

• Thoughts about Diabetes [Personal Models of Diabetes [18,19]: This assesses beliefs about the seriousness of diabetes and treatment effectiveness. It is validated for use with adults with diabetes [20].

• Self-Care Behaviours: The revised Self-Care Inventory, a measure of perceived adherence to diabetes self-care recommendations [21].

• Fear of Hypoglycaemia Questionnaire (Worry subscale; [22]). This scale and the one below assesses the influence of these concerns on self-management and quality of life. Both scales have established validity and reliability in this patient population.

• Fear of Complications Questionnaire [23].

• Social Support Questionnaire [24,25]: The SSQ6 is an abbreviated version of the original 27-item questionnaire. It provides a measure of the number of supportive relationships available and an indication of the level of satisfaction with that support. It has high internal consistency and re-test reliability [26].

• A single-item measure of life satisfaction from the Personal Well-Being Index [27].

• Diabetes Knowledge Questionnaire – Revised Michigan Diabetes Knowledge Questionnaire [28].

• Evaluation of the DAFNE course is assessed at 6 weeks using one, 9-item sub-scale of the Health Education Impact Questionnaire [29].

• Adherence to DAFNE principles: This is assessed at the 6 week, 6 and 12 month follow ups using a DAFNE specific Self-Management questionnaire, developed for the present NIHR Psychosocial study, (MREC 08/H0808/53).

### Analysis

The primary analysis will be a linear model of HbA_1c_ at 12 months with baseline HbA_1c_ as a covariate, (which will improve the power relative to the predicted power), using generalised estimating equations (GEE) to control for clustering within courses. The intracluster correlation coefficient coefficient (ICC) will be estimated using the method of moments. Poisson regression (or a zero-inflated Poisson regression if necessary) will be used on the number of hypoglycaemic episodes over 12 months, which should also have more power than a dichotomy of having/not experiencing an episode, again using GEE to account for clustering. We will use an intention to treat analysis, but will explore the issue of participants dropping out using multiple imputations. The full analysis set (FAS) for the intention to treat analysis includes all patients for whom baseline data was collected. A per protocol analysis will also be performed.

### Health economics analysis

An economic evaluation will be undertaken from the NHS perspective, using both a within trial timeframe and a modelled patient lifetime timeframe. The modelled analysis will be the primary focus of the economic sub-study. Patient costs will be calculated covering training, equipment, drugs and NHS contacts relating to the management of diabetes and its associated conditions. The cost of the training associated with the control and intervention groups will be calculated through a survey of resource use and costs at each of the seven recruiting centres. The survey will cover staff input, consumables, capital and overheads, and will be followed up with discussions with staff at each centre to ensure the quality of information provided.

Patient-level data will be collected for equipment, drugs and NHS contacts. Equipment and drug use will be taken from the trial’s clinical record forms, while the NHS contacts will be taken from the DAFNE Programme’s patient database. This database records all primary care contacts relating to diabetes, specific contacts for hypoglycaemic events, and diagnosis-specific inpatient admissions.

Unit costs will be taken from standard sources (NHS Reference Costs, British National Formulary, and PSSRU). Quality adjusted life years (QALYs) will be estimated using EQ-5D at baseline, 6, and 12 months, and patient mortality.

Two analyses, one within the trial, and another using a lifetime analysis based on the Sheffield Type 1 Diabetes Policy Model (currently in development through the NIHR Programme Grant), will be undertaken. An incremental cost effectiveness ratio (ICER) will be calculated with uncertainty around this characterised by plots on the cost-effectiveness plane and its associated cost-effectiveness acceptability curve. The impact of different training costs as observed in the different centres will be assessed using one-way sensitivity analysis.

### Qualitative sub-study

#### Background

Prior to development of the 5x1 trial, as part of the NIHR programme, a qualitative evaluation had already been undertaken of standard DAFNE courses delivered over one week to understand and explore patients’ likes and dislikes of their courses, and their reasons for following or not following a DAFNE regimen post-course and over time. This qualitative evaluation comprised observation of six DAFNE courses and in-depth interviews with patients (n=30) on completion of their courses and six and 12 months later. Twelve educators were also interviewed. Findings from this work have already been published [30-34] with further papers planned. As we had already collected detailed data on participants’ experiences of, and views about, one week DAFNE courses and their post-course experiences, we decided to focus on the 5 week course participants in the 5x1 trial, and to compare their data to that already collected during the qualitative evaluation of the one week DAFNE courses. The inclusion of this qualitative work in the 5x1 trial follows the NICE recommendation that randomised controlled trials involving diabetes educational interventions should involve a qualitative evaluation of the intervention [35].

### Research questions

1) Are there any differences in the ways in which patients interact with, and support one another, when attending a DAFNE course delivered over 1 or 5 weeks, and why?

2) Are patients more or less inclined and able to make changes to their insulin doses and approach to diabetes management during and after attending a course delivered over 1 or 5 weeks, and why?

3) Do any logistical and/or unanticipated issues arise from the adaptation and delivery of a DAFNE course spread over 5 weeks?

4) If any patients withdraw from the 5 week course, what are their reasons for doing this?

5) What do patients and educators like/dislike about attending/delivering a DAFNE course for one day a week over five weeks? What are the perceived advantages and disadvantages of a DAFNE course delivered over 5 weeks rather than over 1 week? What are patients’ preferences for the timing of DAFNE courses, and why?

6) How can the findings relating to the above research questions be used to explain differences/similarities in biomedical and psychological outcomes between patients attending the two types of courses?

### Study design

1) Observation of five week DAFNE courses to: (a) inform issues and areas explored in interviews with patients and educators who participated in these courses;(b) ascertain, through comparison with observational data collected during the evaluation of standard courses delivered over one week, whether there are any differences between the two types of course (e.g. in terms of style, curriculum delivery, individual and group dynamics); and, (c) identify any unanticipated issues arising from the adaptation and delivery of DAFNE courses over five weeks.

2) Post-course interviews with patients to: explore their experiences of, and views about, the DAFNE course spread over five weeks; any changes they have made to their diabetes management/self-care practices in light of the course (and why); their hopes, expectations and (any) short- and long-term treatment goals set; and (if relevant), reasons for not completing the course.

3) Follow-up interviews with patients at six months to explore whether, and why, they have been able or unable to sustain their skills training in intensive insulin therapy, and any issues which they think may help promote/support effective self-management in the future.

The time-point for follow-up interviews was selected (a) to mirror the design of the qualitative evaluation of the standard courses delivered over one week and (b) because this evaluation had highlighted that, by six months, sufficient time should have elapsed to establish whether, and for what reasons, patients are able/unable to put their skills training into practice and sustain the intensive approach to disease self-management taught on their courses.

To contextualise and enhance understanding of patients’ accounts, other issues were also explored in their initial and follow-up interviews including: work and family commitments and the impact and implications of these for treatment adherence, course attendance, and course preferences etc.; (changing) perceptions and understandings of their disease (e.g. perceived seriousness and controllability); and, their experiences of, views about, and need for health professional and other forms of support prior and subsequent to course attendance.

4) Interviews with educators on completion of courses to reflect upon their understandings and experiences of course delivery; their perceptions and views of (any) differences; issues and/or challenges arising from delivering DAFNE over five weeks rather than over one week; and, recommendations for future course development.

### Data collection

Observational data were recorded in the form of field notes and written up by DR at the end of each day of sitting in on the DAFNE course. Topic guides have been used to inform all interviews to ensure the discussion stays relevant to the study aims and objectives, while allowing participants to raise and discuss issues they perceive as salient to them. These were adapted from those used in the qualitative evaluation of standard DAFNE courses delivered over one week to allow for data comparisons.

Baseline interviews with patients were normally undertaken face-to-face and at a time and location of their choosing (normally in patients’ own homes). Follow-up interviews were undertaken on the phone unless a patient requested a face-to-face interview. Educator interviews were undertaken by phone.

Each patient and educator interview lasted around an hour, and, subject to consent, these were audio-recorded, and transcribed in full to permit in-depth analysis.

### Sample

Four 5 week courses were observed in their entirety, in three trial centres. The sample size of n=30 patients was determined, based on previous experience [36,37], to enable a diversity of perspectives to be examined, a full range of issues to be explored, data saturation to occur (i.e. no new findings emerge from an analysis of new data), and for meaningful comparisons to be drawn between the experiences and views participants on the two types of courses. Initial data collection was staggered over 7 months to allow for issues and findings arising during the early phases of data collection to inform, if necessary, the areas explored in later stages of data collection, in line with an inductive thematic approach.

### Data analysis

Using an ongoing and iterative process data analysis started once the observations and interviews began. The study was informed by the principles of grounded theory [38] and the method of constant comparison [39], which involves concurrent data collection and analysis, together with systematic efforts to check and refine developing categories of data. Data collection stopped in October 2011 and data analysis is currently on-going. Data are being analysed thematically and the two datasets (i.e. one week and five week participant accounts) are being cross-compared to look for differences and similarities in course participants’ experiences, views, course preferences and self-management approaches post course and over time, and the reasons for these. Regular meetings (between JL and DR) are taking place to explore participants’ underlying reasoning, discussion of deviant cases and agreement on recurrent themes and findings. NVivo 9, a qualitative data-indexing package is being used to facilitate coding and retrieval.

## Discussion

A particular strength of this trial is that it has not occurred in isolation, but as one component of a NIHR 5 year programme grant utilising DAFNE as a research test-bed. Professionals from different disciplines have collaborated on other pieces of work within this grant, thus the development of the intervention and conduct of the trial has been informed and influenced by other facets of the research programme. One such element was a psychosocial evaluation of how, and why, in the context of Type 1 diabetes in the UK, structured group education actually works [30-34]. By incorporating qualitative substudies within the programme grant we have aimed to enhance and inform interpretation of findings and recommendations for future delivery of structured education.

The trial has also highlighted some of the problems involved in conducting a non-pharmaceutical funded clinical trial within the NHS. Each centre has relied largely on the goodwill of one or two DAFNE educators, a part-time administrator, plus a consultant physician to recruit participants into the trial and to collect follow-up data. The scale of this latter process cannot be under-estimated, and has been aided in some centres by help from their local CLRN (Clinical Research Network). The programme grant only included a relatively small payment to be made to each centre for running the courses and for entering data (£1250 / course). Development of the five week curriculum, and production of resources was aided by the DAFNE Collaborative.

Data collection is due to be complete by Summer 2012, the results will be analysed by our statistical team, and we hope to publish findings from this randomised controlled trial shortly thereafter.

## Competing interests

The authors declare that they have no competing interest.

## Authors’ contributions

All authors participated in the design of the trial. JE devised the trial protocol and drafted the manuscript. JL and DR devised the psychosocial intervention, DR is conducting the in-depth interviews and both DR and JL are analysing the psychosocial findings. CE helped devise the protocol and is project manager. MJC is lead statistician for the trial. SD is the lead health economist for the trial. SH is CI of the NIHR programme grant for applied research – Improving management of Type 1 diabetes in the UK: the DAFNE programme as a research test-bed. All authors read and approved the final manuscript.

## Pre-publication history

The pre-publication history for this paper can be accessed here:

http://www.biomedcentral.com/1472-6823/12/28/prepub
